# Editorial: Impact of parental education and socio-economic status on children’s oral health behaviors

**DOI:** 10.3389/froh.2025.1755103

**Published:** 2025-12-15

**Authors:** Eman K. M. Mansur, Marilynn L. Rothen, Sherry Shiqian Gao

**Affiliations:** 1 Department of Dental Public Health and Preventive Dentistry, Faculty of Dentistry, University of Benghazi, Benghazi, Libya; 2 Department of Oral Health Sciences, School of Dentistry, University of Washington, Seattle, WA, United States; 3 Department of Stomatology, School of Medicine, Xiamen University, Xiamen, China

**Keywords:** behavior, children, oral health, parental education, socio-economic status

## Introduction

Oral health is a multidimensional concept, shaped by biological, behavioral, social, and structural factors, and represents a fundamental element of overall health and well-being (1). Even though dental caries remains one of the most prevalent oral diseases worldwide, it reflects only one aspect of broader inequalities in oral health. These are influenced by unequal distribution of resources, opportunities, and health awareness (2). Amongst these factors, parental education level and socioeconomic status stand out as key factors that significantly influence the course of oral health in children (3). Families with low levels of education or limited socioeconomic resources often face cumulative obstacles, including low levels of oral health awareness, limited access to preventive dental services, and increased exposure to unhealthy environments (4, 5). These social factors not only shape oral hygiene behaviors and dietary habits, but also determine the family's ability to cope with health systems and adopt preventive practices, thus perpetuating intergenerational inequalities (2, 6).

Despite strong evidence linking parental education, socioeconomic status and oral health outcomes, the mechanisms by which these factors operate are still not adequately covered in the global public health discourse (3). The differences between countries highlight the structural nature of these inequalities, underscoring the need for comprehensive, system-wide strategies to promote health equity (7, 8).

This editorial brings together emerging evidence from diverse settings to illustrate how parental education and socioeconomic status act as overarching determinants of children's oral health. Rather than summarizing individual contributions in isolation, the editorial critically analyzes common themes, identifies persistent knowledge gaps, and considers their implications for public health policy, preventive dentistry, and health equity initiatives.

## Summary of contributions

Across all the included studies, a consistent pattern emerges linking parental knowledge, social support structures, socioeconomic conditions, broader social determinants, and children's oral health outcomes. Taken together, the evidence reinforces the central role of families and contextual factors, as described in conceptual models of oral health inequalities, in shaping caries experience, hygiene behaviors, service utilization, and developmental conditions affecting teeth.

Lally et al., examined 157 parent–child triads attending the Children's Hospital Colorado Dental Clinic, using a cross-sectional design to assess the relationship between social support for Hispanic parents and their children's dental caries. The study concluded that higher levels of parental social support and greater knowledge about using dental services were strongly associated with a lower incidence of caries in children, highlighting the impact of family psychosocial resources on oral health.

Hao et al., analyzed a large retrospective dataset of 16,199 Chinese students aged 7, 9, 12, and 14 years, collected between 2010 and 2019, to study the temporal trends of dental caries prevalence and its relationship to nutritional status. Despite economic progress and improvements in health policies, the study revealed persistently high rates of dental caries, highlighting inequalities consistent with the broader impact of socioeconomic development and access to oral health promotion services. The researchers emphasized the need to integrate oral health and nutrition education into school health programs to reduce preventable inequalities.

Buunk-Werkhoven et al., conducted a cross-sectional study of 420 Lithuanian parents to explore their perceptions of oral hygiene practices and their knowledge of their young children. Although most parents had higher levels of education, the results highlighted a significant gap between knowledge and actual behaviors, particularly regarding supervised brushing and regular dental visits. These findings suggest that parental education alone is insufficient without targeted interventions that support behavior change within evolving public health systems.

Naavaal and Lamsal, used three years' (2017–2019) Medical Expenditure Survey (MEPS) data from 9,927 American children to assess the relationship between children's use of dental services and parental engagement with healthcare services. Their analysis showed that parents who regularly use healthcare services are significantly more likely to ensure their children receive preventive dental care. This intergenerational modeling of health-seeking behavior aligns with established frameworks that emphasize parental influence as a key factor in children's access to preventive care.

Ortega-Luengo et al., investigated the socioeconomic and early nutritional factors associated with molar-incisor hypomineralization (MIH) in children aged 8–16 years at multiple oral health units in Madrid, Spain. This observational cross-sectional study demonstrated strong associations between lower socioeconomic status, suboptimal feeding practices in the first two years of life, and an increased prevalence of MIH. These findings illustrate how early life circumstances shaped by parental knowledge, feeding practices, and socioeconomic constraints contribute to developmental enamel defects and long-term poor oral health.

Finally, Glińska et al., analyzed data from 6,289 adolescents aged 13–15 years from Slovakia and Poland to examine the role of health literacy, family socioeconomic status, gender, age, and country of origin in shaping oral health status and related behaviors. Higher health literacy and family socioeconomic status were strongly associated with better oral health outcomes, while gender-based differences reflected variations in societal expectations and health communication. These findings reinforce the influence of cultural and educational determinants on adolescent oral health and are consistent with global evidence highlighting the interaction between social context and health behaviors.

## Broader context and emerging themes

The studies included in this research topic reveal several interconnected themes, reinforcing the interrelated nature of the determinants of children's oral health. First, parental education and health literacy consistently emerge as key factors influencing children's daily oral hygiene practices, dietary choices, and engagement with preventive dental services. Whether it is social support, knowledge of how to access services, or awareness of appropriate feeding and hygiene behaviors, parental capabilities strongly predict children's oral health outcomes. Second, socioeconomic disadvantage exacerbates barriers to preventive care and healthy behaviors. As low socioeconomic status is associated with higher prevalence of dental caries, suboptimal nutritional practices, reduced access to services, and increased risk of developmental conditions such as MIH. These patterns reflect global evidence showing that inequalities in oral health follow clear social gradients across different countries and health systems. Third, studies highlight the importance of integrated policies and public health interventions, particularly those delivered through schools and family centered programs. Interventions that combine oral health education, nutritional counseling, and structured parental support show promising effectiveness in mitigating inequalities when integrated within broader social and health frameworks.

These findings collectively confirm that oral health is shaped by multi-level interactions involving individual behaviors, family dynamics, community resources, and national policy environments. They also underscore that improving children's oral health requires addressing both structural and behavioral factors simultaneously.

## Implications for research and practice

The body of evidence underscores the need to align oral health promotion with parent-centered educational strategies, particularly during early childhood when health habits and developmental pathways are formed. Clinicians and public health professionals should effectively integrate the social determinants of health into screening, consultations, and care planning, considering parental behaviors and socioeconomic contexts as key elements in risk assessment. Collaboration between healthcare providers, educators, and community organizations is crucial for delivering unified preventative messages and accessible programs, particularly in socially disadvantaged settings. Schools, in particular, are a key platform for integrating oral health promotion with nutritional education and public health awareness initiatives. Regarding future research, studies indicate the need for longitudinal, interventional, and mixed-methods designs to assess the impact of enhancing parental education, improving health literacy, and addressing socioeconomic constraints on tangibly improving children's oral health outcomes. Multidisciplinary approaches, which link dentistry, public health, psychology, behavioral sciences, and education, are essential for addressing the complex social and behavioral roots of oral disease and for designing interventions tailored to high-risk populations.

## Conclusion

This research topic clearly demonstrates that improving children's oral health cannot be achieved through clinical services alone. Sustainable progress requires addressing the educational, behavioral, and socioeconomic foundations that shape oral health from a young age. By integrating parental education, addressing social inequalities, and incorporating preventive strategies into community and school systems, health professionals and policymakers can contribute to reducing disparities and promoting lifelong oral health.
